# Therapeutic Uses of Creasote

**Published:** 1852-02

**Authors:** B. W. Richardson


*On the Therapeutic Uses of Creasote.*—By B. W. Richardson. He stated that during the last visitation of Asiatic cholera his attention had been called to a short notice, communicated to *The Lancet and Medical Gazette*, by Mr. Spinks, of Warrington, on the astringent value of creasote in that disease. Mr. Richardson had never used creasote in true Asiatic cholera, because the subject had not come before him until the epidemic was nearly over, but he had since fully tested its value as an astringent, and he must say, that in some cases of diarrhea, he had never before seen half so good a remedy. The cases in which it is most useful are of three kinds: 1st. Cases of diarrhea during ordinary epidemics where the disorder cannot be traced to the presence of foreign matter in the intestines. 2d. Cases where profuse diarrhea follows the employment of purgatives, given to remove foreign matters during intestinal disorders. 3d. Cases in which, after an acute diarrhea, the patient continues to be troubled with frequent and sudden liquid evacuations, not attended with pain or great constitutional disorder. Illustrative cases were supplied. The advantages of creasote are the following: It often succeeds when all other astringents fail; of this the author is thoroughly convinced from repeated experiments; it is speedy in its action; lastly, it does not leave the bowels constipated, unless carried too far in its administration. Occasionally during its use, it produces a dry, white, filmy state of the tongue, and other symptoms of feverishness. When this occurs the remedy must not be continued longer; indeed, it is rarely required after such symptoms, the purging being usually arrested before they appear. With children the dose given must be very small, or such good results will not follow; the one-eighth, one-sixth, and one-fourth part of a drop, is sufficient for babes from one to two years of age. With adults the dose, as an astringent, is from one and a half to two minims. A late writer in the *Medical Gazette* (Mr. Kesteven) had also alluded to the value of creasote as an astringent, and had opined that this value depended on the power which creasote was known to have of coagulating albuminous fluids. To this it may be objected that the quantities of the creasote employed medicinally are not sufficient to produce such coagulation in the intestines. Mr. Richardson also alluded to the other effects of creasote. He denied that, in ordinary doses, it possessed some of the properties described as belonging to it in elementary treatises on therapeutics, such as narcotic, sedative, and diuretic properties. At the same time he assigned to it powerful diaphoretic and anti-spasmodic qualities, and said, that on the vascular system it rather acted as a stimulant than as a sedative. Its power in stopping vomiting depended upon the dose, which should be small.—Given in its full dose, as an astringent, it sometimes *excites* vomiting, in which case hydrocyanic acid is usefully combined with it. To lessen its nauseous qualities, it is best to unite it with syrup of tolu and tincture of cardamoms, and camphor julep or water. It may also be prescribed advantageously with the preparations of ether when they are indicated.—*London Lancet.*

